# Surprise responses in the human brain demonstrate statistical learning under high concurrent cognitive demand

**DOI:** 10.1038/npjscilearn.2016.6

**Published:** 2016-06-08

**Authors:** Marta Isabel Garrido, Chee Leong James Teng, Jeremy Alexander Taylor, Elise Genevieve Rowe, Jason Brett Mattingley

**Affiliations:** 1 Queensland Brain Institute, The University of Queensland, Brisbane, QLD, Australia; 2 Centre for Advanced Imaging, The University of Queensland, Brisbane, QLD, Australia; 3 ARC Centre for Integrative Brain Function; 4 Centre for Cognitive Neuroscience, Duke-NUS Graduate Medical School, Singapore, Singapore; 5 School of Psychology, The University of Queensland, Brisbane, QLD, Australia

## Abstract

The ability to learn about regularities in the environment and to make predictions about future events is fundamental for adaptive behaviour. We have previously shown that people can implicitly encode statistical regularities and detect violations therein, as reflected in neuronal responses to unpredictable events that carry a unique prediction error signature. In the real world, however, learning about regularities will often occur in the context of competing cognitive demands. Here we asked whether learning of statistical regularities is modulated by concurrent cognitive load. We compared electroencephalographic metrics associated with responses to pure-tone sounds with frequencies sampled from narrow or wide Gaussian distributions. We showed that outliers evoked a larger response than those in the centre of the stimulus distribution (i.e., an effect of surprise) and that this difference was greater for physically identical outliers in the narrow than in the broad distribution. These results demonstrate an early neurophysiological marker of the brain’s ability to implicitly encode complex statistical structure in the environment. Moreover, we manipulated concurrent cognitive load by having participants perform a visual working memory task while listening to these streams of sounds. We again observed greater prediction error responses in the narrower distribution under both low and high cognitive load. Furthermore, there was no reliable reduction in prediction error magnitude under high-relative to low-cognitive load. Our findings suggest that statistical learning is not a capacity limited process, and that it proceeds automatically even when cognitive resources are taxed by concurrent demands.

## Introduction

Learning about regularities in the world is a fundamental ability of adaptable animals. This ability to predict what will happen next provides a competitive advantage for anticipating reward or avoiding punishment. Both we and others have previously demonstrated that the human brain can implicitly learn about, and detect, violations in these regularities.^1–3^ A large number of studies investigating regularity learning have employed auditory oddball paradigms in which so-called ‘standard’ sounds establish a given rule, or a prediction, and occasional ‘oddball’ events violate that rule. These prediction violations evoke a conspicuous early brain response (peaking at about 100–250 ms), which can be recorded with magneto- and electroencephalography (M/EEG). This response, called the mismatch negativity (MMN), is thought to reflect a sensory prediction error and a neurophysiological marker of a learned regularity.^4,5^

In the classic, and most commonly used, oddball paradigm, standard sounds are repeated single pure-tone events and oddballs are rare sounds that differ from standards in some physical property such as frequency,^6–9^ duration^10^ or amplitude.^11^ More sophisticated paradigms have employed abstract regularities, albeit based on deterministic sequence-based rules, such as a sequence of regularly descending tone pairs broken by an occasional ascending combination.^12^ In contrast, we have designed a novel paradigm in which the regularity is completely probabilistic: it can not be encoded as a deterministic sequence-based rule, a finite set of known stimuli, or by a categorical separation between expected and unexpected events.^1^ In that study, we showed that people can implicitly learn statistical patterns while performing a simple incidental detection task, and that the strength of the MMN depends on the relative likelihoods of oddball stimuli. In more ecological scenarios, however, learning about regularities will often occur in the context of competing cognitive demands that may impose limits on cognitive resources.

It has been shown that sensory prediction error responses can be elicited when cognitive resources are taxed by concurrent demands, for example, while people perform challenging tasks^13–15^ and even during non-conscious states such as sleep^16^ or coma.^9,17^ This demonstrates the brain’s capacity to detect changes automatically,^18^ even when cognitive resources are limited. These studies have typically employed rule violations simply evoked by a change occurring after several repetitions of the same event. While such observations suggest that the brain can detect simple changes when voluntary attention is reduced or absent, it is unclear whether the system can implicitly learn, and detect violations to, complex patterns while performing a cognitively demanding task. Here we asked whether such learning can occur, and if so, whether it is compromised by depletion in cognitive resources associated with performance of a concurrent task. To address these questions we used our novel MMN paradigm,^1^ which employs a probabilistic regularity, in conjunction with a working memory task to tax cognitive resources.^19^ We hypothesised that the brain should be able to learn, and detect violations to, statistical regularities even while performing a highly demanding cognitive task.

## Results

### Single-channel analysis

**Figure 1:**
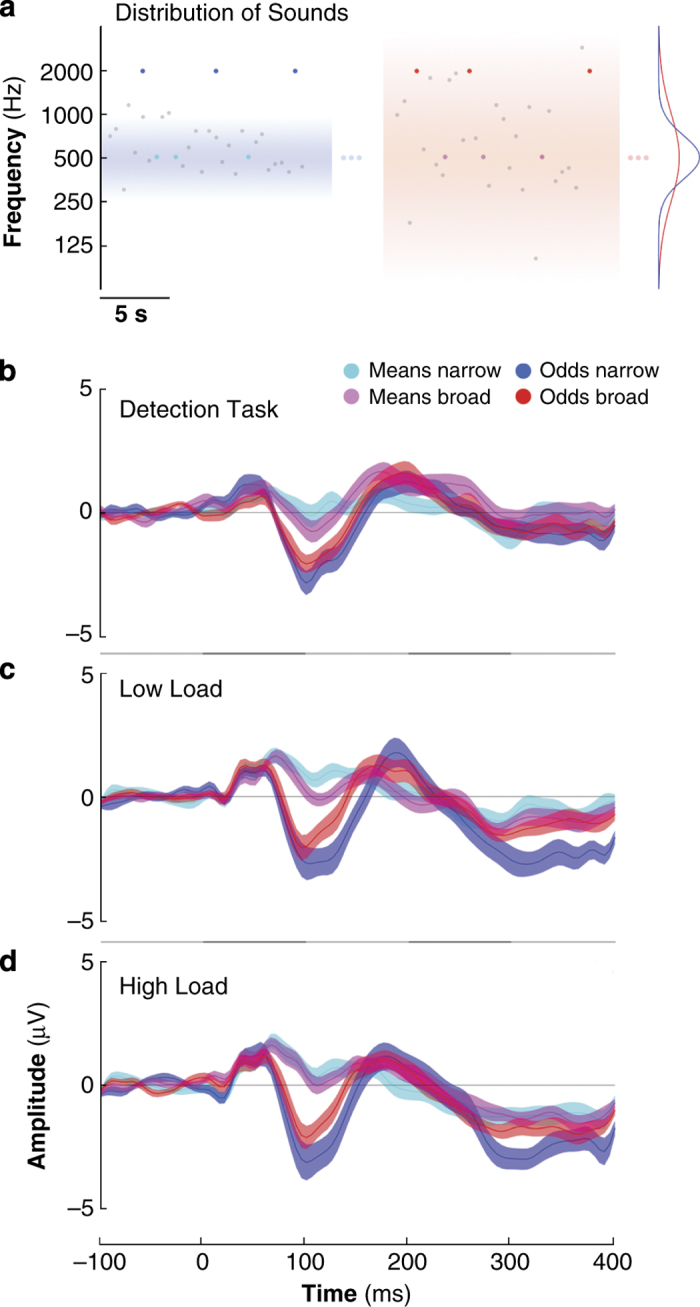
Stimulus distributions and prediction error responses. (**a**) Stimuli presented in Experiments 1 and 2. The frequencies of the majority of pure-tone sounds in each block (grey) were drawn from a contextual distribution that could be narrow (left, blue shading) or broad (right, red shading). The distribution densities are shown on the right in blue and red shading; both were centred at 500 Hz and had s.d. of 0.5 and 1.5 octaves, respectively. Embedded in both sequences were probe tones whose frequencies were either equal to the distribution centres (means, cyan and magenta), or 2 octaves above (odds, blue and red). (**b**–**d**) Brain responses evoked by mean and oddball sounds in the context of the narrow and the broad distributions, recorded in a fronto-central channel (FCz) while participants performed a detection task (Experiment 1, **b**) and a working memory task (Experiment 2) with low (**c**) and (**d**) high cognitive load.

Our goal was to demonstrate that the brain is able to learn, and detect violations to, statistical regularities even under high concurrent cognitive demands. To do so, we recorded EEG data while human participants listened to a stream of pure-tone sounds sampled from narrow and broad Gaussian distributions centred at a given frequency (see Figure 1a). We first examined whether our results replicated the MEG findings reported by Garrido *et al.*,^1^ which employed the same paradigm as used here in Experiment 1; that is, the same sound structure (as illustrated in Figure 1a) was played while participants performed a simple incidental visual detection task. We contrasted evoked responses to odds and means under narrow and broad distributional variance (event-related potentials (ERPs) recorded at fronto-central channel FCz, displayed in Figure 1b), and indeed found that responses to odds were larger than responses elicited by means, regardless of contextual variance and within the typical MMN time window (main effect of surprise, F(1,18)=46.71, *P*=2.14×10^−6^). In addition, we found that these MMNs, or prediction error responses, were larger under the narrow than the broad distribution (surprise by variance interaction, F(1,18)=7.99, *P*=0.011). Follow-up analysis revealed larger responses to odds within the narrow than the broad distribution conditions (*t*(18)=−2.32, *P*=0.032), but no differences were found for means between the narrow and the broad contexts overall (*t*(18)=1.43, *P*=0.169). These results replicate our previous MEG findings (Garrido *et al.*^1^), and demonstrate that the brain is sensitive to outliers and to contextual uncertainty.

Next, in Experiment 2, we again used the same sound structure but manipulated cognitive demands by adding a secondary task at fixation that imposed a working memory load (*N*-back) with two levels of difficulty (low and high cognitive load; 1-back versus 2-back). In this task, participants were significantly slower (F(1,16)=14.91, *P*=0.001) and less accurate (F(1,16)=29.89, *P*=5.17×10^−5^) in the high-load than in the low-load condition, regardless of contextual variance, thus verifying the effectiveness of the cognitive load manipulation. For the incidental auditory streams, which were identical to those employed in Experiment 1, we again found larger responses to odds than to means, regardless of cognitive load (main effect of surprise, F(1,16)=43.02, *P*=7×10^−6^) and this difference was again larger under the narrow than the broad distribution (surprise by variance interaction, F(1,16)=22.83, *P*=2.05×10^−4^, see Figure 1c,d). These results demonstrate that the brain is sensitive to outliers and contextual variance even when it is engaged in a cognitively demanding task.

### Spatiotemporal analysis

**Figure 2:**
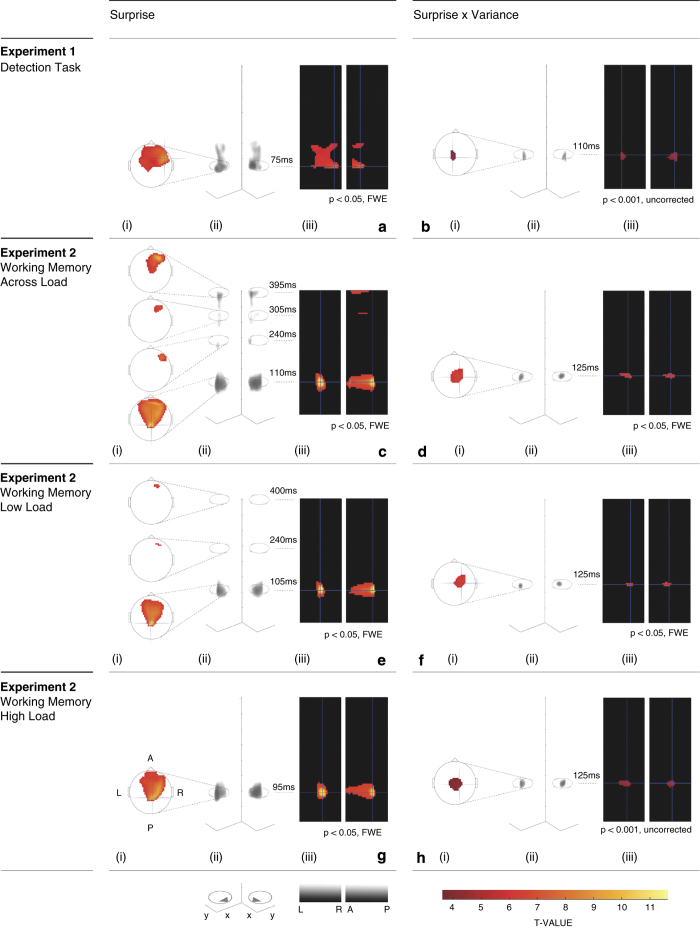
Spatiotemporal maps reveal statistical learning under cognitive load. Spatiotemporal statistical analysis revealed significant effects of surprise (left column) and surprise–variance interaction (right column) over fronto-central areas at early time points (about 100 ms) for both the detection task (Experiment 1, **a** and **b**) and the working memory task (Experiment 2, **c**–**h**). (**a**) Results for the detection task (Experiment 1). Main effect of surprise (odds>means) and (**b**) the interaction between surprise and variance (odds versus means larger in the narrow versus the broad distribution. Results for the working memory task (Experiment 2). (**c**) Main effect of surprise and (**d**) the interaction between surprise and variance regardless of cognitive load. (**e**) Simple effect of surprise and (**f**) the interaction between surprise and variance for the low-cognitive load condition. (**g**) Simple effect of surprise and (**h**) the interaction between surprise and variance for the high cognitive load condition. (i) 2D scalp topographic maps at peak statistical difference per cluster; (ii) 3D representation of responses with spatial dimensions on *x–y* plane, time domain along *z*-axis and views from dual angles; (iii) Statistical parametric map at peak statistical difference, anterior–posterior (A–P) and left–right (L–R) sectional views.

The fine temporal resolution and spatial coverage afforded by the high-density EEG recordings permitted a whole-volume spatiotemporal analysis. We converted the whole data into three-dimensional (3D) spatiotemporal images (see Methods for further details) and modelled these data with a GLM approach as typically used in functional magnetic resonance imaging studies. As above, we employed analysis of variance (ANOVA) designs to investigate effects of surprise, variance and cognitive load. With this approach, however, we could fully explore the richness of the whole data set in an unbiased way, that is, with no *a priori* assumptions about when in time and in which particular channel our effects might be. In Experiment 1, we found a large cluster for the main effect of surprise, which peaked at 75 ms and spread over a fronto-central scalp region (*P*<0.05, family-wise error (FWE) corrected, Figure 2a). In addition, we found one cluster for the interaction of surprise and variance, which peaked at a central region of the scalp at 110 ms (Figure 2b, statistical map displayed at *P*<0.001 uncorrected). This cluster reached significance with a small-volume correction (SVC, FWE-corrected *P*<0.014) for the reduced volume of interest, based on the orthogonal main effect of surprise (shown in Figure 2a). These results are consistent with our previous findings,^1^ and demonstrate the brain’s sensitivity to outliers, as well as its ability to learn about the statistical structure of sensory events.

In Experiment 2, we asked whether the ability to learn the statistical structure of the tones was preserved or modulated by concurrent cognitive demands, as manipulated by the *N*-back working memory task. We found that responses to odds were larger than responses to means, regardless of distributional variance and cognitive load, again at fronto-central regions of the scalp and peaking as early as 110 ms, as well as at later time points (*P*<0.05 FWE-corrected, Figure 2c). The interaction between surprise and variance, regardless of load, resulted in a central cluster peaking at 125 ms (*P*<0.05 FWE-corrected, Figure 2d), which is very similar in spatial coverage and temporal profile to the effect found for the same interaction in Experiment 1. In addition, we found a number of clusters for the effects of load and the three-way interaction (but only at *P*<0.01 uncorrected) over occipital-parietal regions, but these failed to reach significance when corrected for multiple comparisons. This shows that the human brain can indeed learn about, and detect changes to, statistical regularities, even while occupied with a cognitively demanding concurrent task.

Finally, we conducted follow-up tests to determine whether learning ability was preserved in either, or both, cognitive load conditions (Figure 2e–h). We found a main effect of surprise in both load conditions, with a common fronto-central cluster peaking at about 100 ms (105 ms and 95 ms for high load and low load, respectively, both at *P*<0.05 FWE-corrected; compare Figure 2e,g). Under low load, however, we found two extra frontal clusters at later time points (peaking at 240 and 400 ms). The interaction of surprise by variance yielded one early cluster peaking at 125 ms in both load conditions, although this cluster was less significant under the high-load condition (displayed at *P*<0.05 FWE-corrected and *P*<0.001 uncorrected, respectively; compare Figure 2f,h). In the low-load condition, the significant cluster survived whole-volume FWE correction and was slightly right lateralised, whereas in the high-load condition there was an early central cluster, *P*<0.01 FWE (for the volume defined in the orthogonal effect of surprise under high load, shown in Figure 2g).

## Discussion

In this study, we investigated the role of concurrent cognitive demands on learning about statistical relationships in the auditory sensory environment. In two separate experiments, we had participants perform a visual task while listening to an apparently random sequence of sounds, which, unbeknownst to them, was governed by a specific statistical structure. Sounds were drawn from two normal distributions with equal mean frequencies and distinct contextual variances: narrow and broad. We examined sensory prediction error responses with EEG by contrasting outliers and mean sounds under both narrow and broad variance contexts and found that outliers evoked a larger response than mean sounds. Moreover, we found that this prediction error response was larger for the narrow than broad distribution, a context under which outliers were less likely. Importantly, these findings were robust in both experiments, that is, when participants performed a simple visual change detection task (Experiment 1, and in keeping with our MEG findings^1^), and also when they were engaged in a visual working memory task (*N*-back; Experiment 2). Crucially, our findings demonstrate that people are able to implicitly learn about, and detect violations to, statistical relationships in the sensory environment even when engaged in a demanding concurrent task.

We had two principal goals in conducting this study. First, we aimed to replicate the findings from our previous study,^1^ which involved an identical paradigm to that used here in Experiment 1, but in which neural activity was measured using MEG instead of EEG. Importantly, we successfully replicated our MEG findings of larger neurophysiological responses evoked by outliers (compared with means), which were larger if these outliers were more unlikely. This provides further support to the idea that the brain is sensitive to statistical structure in the environment.^1,2^ It is important to note that the greater MMN-like response we observe when comparing outliers with sounds at the centre of the distribution is most likely caused by a combination of two processes: a release from adaption^20^ after a change in the stimulus, and a second-order memory comparison mechanism.^5,8^ In our previous study,^1^ we demonstrated that while release from adaptation plays an important role in the generation of prediction error responses, it does not account for them entirely. Indeed, it appears that a mechanism of sensory learning and comparison between inputs and a memory trace may be engaged above and beyond simple adaptation.

In addition, by manipulating the cognitive load imposed by a concurrent working memory task, we were able to show that such learning proceeds even when cognitive resources are heavily taxed. We did not find a significant relative difference in the surprise effect for the low- and high-cognitive load conditions, despite verifying that the visual load effect was indeed effective in that participants were both slower and more error prone in the high-load than in the low-load condition. There was, nevertheless, a clear difference (by visual inspection) in the significance maps for prediction errors (main effect of surprise and surprise by variance interaction) under both low- and high-cognitive loads. However we wish to highlight the need for caution in interpreting the absence of an interaction effect, which might simply reflect the rather strict correction for multiple comparisons under random field theory, a lack of power or both. To examine these possibilities, we used MarsBaR toolbox^21^ (http://marsbar.sourceforge.net/) to compute the effect size of the load by surprise interaction effect based on a cluster extracted from an uncorrected map thresholded at *P*<0.001, which resulted in a value of 1.06. A power analysis based on 0.80 and 0.95 power and an *α* of 0.0001 suggests we would need 26 and 33 participants, respectively, to find a reliable effect (we were left with 17 participants after applying relevant exclusion criteria). The choice of *α* is not trivial since it should be comparable to a corrected threshold, which is hard to compute. Our choice of 0.0001 is based on the similarity between the *P*<0.05 FWE-corrected and *P*<0.0001 uncorrected maps for the interaction of variance and surprise. Hence, if this effect were real, to find it we would need to run our experiment again with at least 26 participants.

We also performed a Bayesian analysis^22^ to compare the posterior probability of a Null model, that is, a model without a load by surprise interaction, with a model including this interaction. The Null model included two regressors, one for the effect of surprise and the other for the interaction between surprise and variance, each of which had revealed significant effects in the standard SPMs. The Load×Surprise model included the same regressors as the Null model, plus a third regressor that modelled the putative interaction between surprise and load. We fit these two models to the whole spatiotemporal scalp ERP data and obtained probability maps for each model over the whole space and time. Our Bayesian analysis revealed that overall, the Null model explained the data better than the Load×Surprise model, particularly at the time points where we found clusters above *P*<0.05 uncorrected. However, the Load×Surprise model won at an earlier time point (between 70 and 130 ms), which was not significant for the load by surprise interaction, even in the uncorrected *P*<0.05 maps. Hence, while our Bayesian analysis suggests that the absence of a load by surprise interaction is the most likely explanation for our data, it suggests there might be an interaction at an earlier time point that was not uncovered by the mass-univariate approach.

The absence of a cognitive load effect on learning would seem inconsistent with cognitive load theory, which postulates that the brain has limited cognitive resources.^23^ A sensible prediction of this theory is that taxing working memory should leave fewer cognitive resources for learning about auditory regularities, which in turn would lead to smaller prediction errors. In contrast, we found that prediction errors were statistically indistinguishable under low and high cognitive loads. The absence of any modulation of prediction errors by cognitive load—specifically working memory load in this case—is in keeping with the idea of automaticity in the brain’s change detection system,^18^ and of the MMN in particular (consistent with SanMiguel *et al.*^24^; but see Lv *et al.*,^15^ who found an increase in the MMN with cognitive load). Caution is needed, however, in interpreting the lack of a cognitive load effect in our data. There are a number of important differences between our paradigm and those of Lv *et al.*^15^ and SanMiguel *et al.*,^24^ who both found reduced P300 amplitude with working memory load. First and foremost, the unpredictability of our odd sounds was conditional on participants learning—probably implicitly—about the statistical relationships among the auditory stimuli. On the other hand, the above-mentioned studies used environmental sounds such as those produced by a drill, hammer, telephone ringing, etc., as oddballs, and pure tones for standards. In that context, standard and oddball sounds were physically very different from each other, and importantly, oddballs had a much higher intrinsic behavioural salience in their paradigm than in ours, which might have engaged other cognitive associations and neuronal circuits. Hence, the ‘oddball-ness’ of our outliers was of a fundamentally different nature to those deviants employed by Lv *et al*.^15^ Another important point of divergence between the paradigms is that in our implementation the working memory load depleted cognitive resources in a continuous manner, i.e., for practically the entire duration of each trial. By contrast, in Lv *et al.*^15^ and SanMiguel *et al.*,^24^ this manipulation was done prior to (instead of during) the presentation of sounds and on a trial-by-trial basis.

Our findings are consistent with those of Bekinschtein *et al.*,^25^ who proposed the notion of local and global (sequence-based) rules and found early (MMN) signatures of local rule violation, even when people performed a working memory (letter detection) task. Indeed, our paradigm established a (non-sequential) statistical regularity with local violations reflected in early neurophysiological responses such as MMN. In contrast to Bekinschtein *et al.*,^25^ however, we also found markers of global violation at early time points (at MMN latency) as shown in the surprise by variance interaction contrasts. In the paper by Bekinschtein *et al.*^25^, neurophysiological markers of global violations were only found in later time periods and only if participants were aware of the regularity structure. In our study, however, participants reported having no awareness of the relationship among sounds. In light of our data, and contrary to Bekinschtein *et al.*,^25^ conscious awareness of regularity does not seem to be necessary for a neuronal signature of global rule violation.

It is worth noting that we chose to manipulate task load in the visual modality while statistical learning was probed in the auditory domain. This choice was driven by an attempt to extract cleaner auditory evoked responses. The question remains, however, as to whether we would find an interaction between cognitive load and learning, had both processes occurred in the same sensory modality and competed for common attentional resources. Indeed, it is plausible that cognitive resources are independently allocated to different modalities,^26^ and hence no cross-modality interference would be observed. In other words, taxing visual resources may have no impact on auditory resources or learning. An alternative explanation is that our task manipulation did not push the working memory system to its full capacity, which may have left spare capacity available for both implicit auditory learning and the visual task, and hence concealed a potential cross-modal interference (ref. 27, but see ref. 28). This cross-modal interference might have been present, had we taxed the system even further with higher load (i.e., *N>*2*-*back task) or with a different working memory task. Verbal working memory, for example, shares common networks with those engaged by auditory sensory learning. These two processes might therefore compete for a common pool of resources if they occur concurrently.

In this study, we manipulated the cognitive load of the task at hand. It is worth reflecting on the possible consequences of manipulating perceptual (instead of cognitive) load. Perceptual load theory^29^ posits that processing of goal-irrelevant stimuli depends on the type and level of load on the task at hand, decreasing with perceptual task load and, conversely, increasing with cognitive task load. In a future study it would be interesting to manipulate perceptual load for the central visual task and to investigate its effect on statistical learning. In light of perceptual load theory^29^ it seems reasonable to predict a decrease in learning, and hence a smaller surprise effect, evoked by goal-irrelevant outliers.

Given the unequivocal evidence that people can learn about statistical regularities in the environment even while engaged in a demanding cognitive task, it is tempting to speculate that such learning may remain unaffected in clinical conditions in which cognitive (working memory) or attentional capacities are compromised, such as in individuals with attention-deficit/hyperactivity disorder,^30^ or those with attention deficits (e.g., unilateral neglect) due to stroke.^31^

In sum, by investigating sensory prediction errors with EEG we have demonstrated that people can implicitly learn about statistical patterns in the auditory environment even when taxed by a concurrent, cognitively demanding visual task.

## Materials and methods

### Ethics statement

Informed consent from each participant was obtained after a full explanation of the experiment, according to the procedures approved by The University of Queensland Medical Research Ethics Committee.

### Participants

EEG data were recorded from 41 adult participants in 2 experiments (20 in Experiment 1 and a separate pool of 21 participants in Experiment 2). We excluded 1 participant in Experiment 1 due to excessive noise in the EEG signal, and hence analysis was conducted on a total of 19 participants (10 males, age range: 19–46 years, mean age: 24 years). In Experiment 2, we excluded 1 participant due to a technical failure and 3 participants due to underperformance (hit rate <50%) on the load task (11 males, 6 females, age range 19–30 years, mean age 23 years). All participants were healthy volunteers, had no known history of psychiatric or neurological disorders or previous head trauma, resulting in unconsciousness, and had normal or corrected-to-normal vision. Participants were naive to the study and were compensated financially for their time. After the experiment, the participants were debriefed about whether they noticed anything particular about the sounds. None of the participants reported being aware of a pattern in the sounds.

### Experimental design

In both Experiments 1 and 2, participants listened to a stream of sounds sampled from two Gaussian distributions with equal means and different variances (narrow and broad, displayed in blue and red shades in Figure 1a) while performing a visual detection task (Experiment 1) or a working memory task (Experiment 2). The auditory stream consisted of a series of pure tones with frequencies sampled from a Gaussian distribution (in log-frequency) centred at 500 Hz, with a s.d. of either 0.50 octaves (narrow variance condition) or 1.50 octaves (broad variance condition). All tones had identical durations of 50 ms, a smooth rise and fall period of 10 ms, and were presented every 500 ms at the same comfortable volume throughout the experiments. Two types of probe tone, equivalent to the standard and oddball events in classic MMN paradigms, were embedded within the tone streams pseudo-randomly, with frequencies equal to the standard (mean probes: *f*_s_=500 Hz, cyan/magenta) or two octaves higher (oddball probes: *f*_o_=2000 Hz, blue/red). These probe tones were indistinguishable from those drawn from the Gaussian distribution. The resulting distribution of frequencies was not strictly Gaussian as each probe type (mean/oddball) was presented 10% of the time, while the Gaussian stream made up the remaining 80% of frequencies. This was necessary to ensure a good signal-to-noise ratio comparable to that typically found in MMN studies. Since only the width of the auditory stream’s frequency distribution differed, ERP comparisons were made based solely on responses to the probe tones.

In Experiment 1, we employed the exact same paradigm first described in our previous study.^1^ Participants were asked to detect luminance changes in a fixation cross displayed on a screen via a key press, while ignoring the concurrent auditory stream. The fixation cross had two levels of luminance, which changed at random intervals between 2,000 and 5,000 ms (in increments of 500 ms). The experiment was presented in two blocks of 13 min each for the narrow and broad distribution conditions. Blocks were counterbalanced across participants. There were ~160 trials of each type (mean or oddball), in each block (narrow or broad) and for every participant.

In Experiment 2, participants monitored a stream of letters presented in the centre of a computer display, and were asked to perform a visual *N*-back task while ignoring the sounds. The *N*-back task required participants to press a key when the currently presented letter matched the letter that was presented either one (low-load condition) or two letters (high-load condition) prior to the current letter. This formed the basis of the cognitive load manipulation. Each letter was displayed for 400 ms, with an inter-stimulus interval of 1,000 ms, plus a randomly determined temporal jitter of between 0 and 500 ms. The experiment was presented in 16 counterbalanced blocks of 210 s each (4 blocks per condition: low- and high-load under narrow and broad variance distributions).

The experimental procedures and stimuli were written in MATLAB (Mathworks, Natick, MA, USA) and the Cogent 2000 toolbox (London, UK).

### EEG recording and preprocessing

Continuous EEG and EOG data were acquired using a BioSemi Active Two 64 Ag-AgCl electrode system (BioSemi, Amsterdam, Netherlands). Electrodes were attached according to the international 10–10 system for electrode placement^32^ and standard BioSemi reference electrodes were used to reference all scalp electrode channels. Data were sampled at 1,024 Hz, with 24-bit A/D conversion and a 0.16–100 Hz amplifier band pass. Offline processing was applied using Statistical Parametric Mapping (SPM8) software (Wellcome Trust Centre for Neuroimaging, UCL, London, UK). Experimental trials were epoched from −100 ms to +400 ms in peri-stimulus time. Data were then downsampled from 1,024 to 200 Hz, filtered using two applications of a Butterworth filter at 0.50 and 40 Hz, thresholded at 100 μV for artefacts, and baseline corrected between −100 and 0 ms.

### Spatiotemporal image conversion

Averaged ERP data were converted into 3D spatiotemporal images for each participant. A 2D 32×32 matrix was produced per time bin, and the images were then stacked according to their peri-stimulus temporal order. This resulted in a 3D spatiotemporal image volume (32×32×101) per participant. These images were smoothed at full width half maximum of 8 mm×8 mm×20 ms.

### Statistical analysis

#### Single-channel analysis

Statistical analyses were performed for channel FCz, a fronto-temporal channel in which MMN responses elicited in oddball paradigms are typically recorded.^18,33^ Mean ERP values were obtained by averaging across a preselected time window of interest and taken over to ANOVA models. In Experiment 1, we had a 2×2 ANOVA design with factors Surprise (odds, means) and Variance (narrow, broad), and in Experiment 2 we had a 2×2×2 ANOVA design with the extra factor for Cognitive Load (low, high). To ensure an unbiased selection for the time window of interest, we computed the mean ERP across all conditions (four conditions in Experiment 1 and 6 in Experiment 2) and chose the first two time points where the ERP crossed the zero line (after stimulus onset). With this procedure, we obtained windows of interest that overlap with typical MMN latencies,^33^ namely 80–150 ms in Experiment 1 and 55–165 ms in Experiment 2. Note that this choice is orthogonal to the statistical tests performed.

#### Spatiotemporal maps

We modelled the 3D spatiotemporal image volumes with a mass-univariate general linear model approach as implemented in SPM.^34^ Data were modelled with one regressor per condition. In Experiment 1, we had a 2×2 design with surprise and variance as factors and four regressors: means and oddballs under the narrow and broad variance conditions. In Experiment 2, we had the extra factor of cognitive load resulting in a 2×2×2 design with eight regressors: means and oddballs under narrow and broad variance, and in the low and high cognitive load conditions. We estimated full factorial ANOVA models for each participant and computed contrast images for main effects and interactions. We then carried these contrasts over to a one-sample *t*-statistic and assessed the significance of these tests across the group. This approach enables an unbiased statistical inference over the whole 3D spatiotemporal data space (2D sensor space and time). Unless stated otherwise, these statistical maps are reported at a threshold of *P*<0.05, with a FWE correction for multiple comparisons for the whole spatiotemporal volume.
